# Heat shock protein 83 plays pleiotropic roles in embryogenesis, longevity, and fecundity of the pea aphid *Acyrthosiphon pisum*

**DOI:** 10.1007/s00427-016-0564-1

**Published:** 2016-10-14

**Authors:** Torsten Will, Henrike Schmidtberg, Marisa Skaljac, Andreas Vilcinskas

**Affiliations:** 1 Institute of Insect Biotechnology, Justus-Liebig-University of Giessen, Heinrich-Buff-Ring 26-32, 35392 Giessen, Germany; 2 Institute of Phytopathology, Justus-Liebig-University of Giessen, Heinrich-Buff-Ring 26-32, 35392 Giessen, Germany; 3 Fraunhofer-Institute for Molecular Biology and Applied Ecology (IME) Project Group ‘Bioresources’, Winchesterstraße 2, 35394 Giessen, Germany

**Keywords:** *Acyrthosiphon pisum*, HSP83, HSP90, Longevity, Fecundity, Development, Viviparous reproduction, Epigenetics

## Abstract

Heat shock protein 83 (HSP83) is homologous to the chaperone HSP90. It has pleiotropic functions in *Drosophila melanogaster*, including the control of longevity and fecundity, and facilitates morphological evolution by buffering cryptic deleterious mutations in wild populations. In the pea aphid *Acyrthosiphon pisum*, HSP83 expression is moderately induced by bacterial infection but upregulated more strongly in response to heat stress and fungal infection. Stress-inducible heat shock proteins are of considerable evolutionary and ecological importance because they are known to buffer environmental variation and to influence fitness under non-optimal conditions. To investigate the functions of HSP83 in viviparous aphids, we used RNA interference to attenuate its expression and studied the impact on complex parameters. The RNA interference (RNAi)-mediated depletion of HSP83 expression in *A. pisum* reduced both longevity and fecundity, suggesting this chaperone has an evolutionarily conserved function in insects. Surprisingly, HSP83 depletion reduced the number of viviparous offspring while simultaneously increasing the number of premature nymphs developing in the ovaries, suggesting an unexpected role in aphid embryogenesis and eclosion. The present study indicates that reduced HSP83 expression in *A. pisum* reveals both functional similarities and differences compared with its reported roles in holometabolous insects. Its impact on aphid lifespan, fecundity, and embryogenesis suggests a function that determines their fitness. This could be achieved by targeting different client proteins, recruiting distinct co-chaperones or transposon activation.

**Electronic supplementary material:**

The online version of this article (doi:10.1007/s00427-016-0564-1) contains supplementary material, which is available to authorized users.

## Introduction

Heat shock proteins (HSPs) are evolutionarily conserved chaperones whose predominant function is to prevent the misfolding and denaturation of proteins caused by environmental stressors such as heat, toxins, or pathogens (Johnson 2012). Their functions in *Drosophila melanogaster* are associated with the buffering of environmental variations, determining fitness under non-optimal conditions, and are therefore of significant evolutionary and ecological relevance (Sorensen et al. 2003). HSPs have been assigned to five families based on homology and molecular mass. The HSP90 family is particularly relevant in the context of evolutionary biology because one member (HSP90) acts as a capacitor for morphological evolution in *D. melanogaster* (Rutherford and Lindquist 1998) by buffering phenotypic variance producing altered phenotypes in response to environmental stressors. The silencing of HSP90 generates variation by transposon-mediated “canonical” mutagenesis (Specchia et al. 2010).

The pleiotropic roles of HSP90 family members in *D. melanogaster* are associated with spermatogenesis, oogenesis, and embryogenesis (Ding et al. 1993; Yue et al. 1999; Song et al. 2007; Pisa et al. 2009) as well as the buffering of cryptic deleterious mutations in wild populations, longevity, and fecundity (Chen and Wagner 2012). In the beetle *Tribolium castaneum*, another holometabolous model insect, HSP83, which belongs to the HSP90 family, is expressed in the whole body as well as in the oocytes where it is specifically located in the follicle cells. There it is differently expressed during different stages of oogenesis (Xu et al. 2010) and in response to heat shock (Xu et al. 2009). The latter suggests that HSP90 family members may regulate physiological processes in response to, e.g., environmental signals (Erlejman et al. 2014). In the whole body of *T. castaneum*, the expression of HSP90 reaches its highest levels during the larval and pre-pupal phases and the attenuation of its expression negatively affects compound eye development in larvae, suggesting that members of the HSP90 family are essential for normal post-embryonic development (Knorr and Vilcinskas 2011). A phylogenetic analysis of arthropod HSP90 genes reveals that the sequences cluster according to their taxonomic order, with holometabolous and hemimetabolous species showing clear separation (Knorr and Vilcinskas 2011).

In response to heat shock, the hemimetabolous whitefly *Bemisia tabaci* shows no differential expression of members of the HSP90 family (Lü and Wan 2011). In the aphid species *Acyrthosiphon pisum*, the first hemimetabolous insect with a completely sequenced genome (The International Aphid Genomic Consortium 2010), Gerardo et al. (2010) demonstrated that HSP83 expression is induced fivefold in response to heat stress. The latter study also showed only minor differences in expression levels between untreated controls and aphids exposed to environmental stress or pathogens. To determine whether HSP90 family members show overlapping or diverse functions in holometabolous and hemimetabolous insects, we investigated the direct impact of HSP83 expression on reproduction in the pea aphid *A. pisum*, as previously shown for HSP90 in *D. melanogaster* (Chen and Wagner 2012). Aphids have evolved complex life cycles including the alternation of sexual and asexual reproduction, with an unusual (autosome-like) inheritance of the X chromosome (The International Aphid Genomic Consortium 2010).

The attenuation of gene expression by RNA interference (RNAi) is a powerful method for the functional analysis of genes in *A. pisum* (Mutti et al. 2006; Jaubert-Possamai et al. 2007; Will and Vilcinskas 2013). We therefore attenuated HSP83 expression in viviparous *A. pisum* by microinjecting the aphids with the corresponding double-stranded RNA (dsRNA). Several fitness parameters were observed in the injected insects to determine the effect of HSP83 attenuation on longevity, fecundity, and embryogenesis.

## Material and methods

### Aphid and plant rearing

The rearing of *A. pisum* clone LL01 and the cultivation of the host plant *Vicia faba* var. *minor* were carried out as previously described (Will and Vilcinskas 2015). During the experiments, aphids were kept on detached, mature *V. faba* leaves under controlled environmental conditions (Mutti et al. 2006; Will and Vilcinskas 2015).

### *RNAi-*mediated attenuation of *HSP83* expression

The RNAi-mediated suppression of HSP83 expression was carried out as previously described (Will and Vilcinskas 2015). Briefly, the Ambion MEGAscript T7 Kit (Applied Biosystems, Austin, TX) was used to prepare dsRNA according to the manufacturer’s protocol. Gene-specific primers including the T7 polymerase promoter sequence at the 5′ end were used to synthesize a 530-bp HSP83 (GenBank XM_001943137.3) dsRNA template (forward primer 5′-TAA TAC GAC TCA CTA TAG GGA GAG TGA GCC GCA TCA AGC CTA AC-3′, reverse primer 5′-TAA TAC GAC TCA CTA TAG GGA GAT ATC AGC CTC GGC CTT CTG TC-3′). We excluded the presence of sequence overlaps >19 bp with other *A. pisum* genes to avoid off-target effects. The QIAquick PCR Purification Kit (Quiagen, Hilden, Germany) was used for template preparation, and dsRNA was produced using the Ambion MEGAscript RNAi kit (Applied Biosystems). Primers were designed with Primer3 (Rozen and Skaletsky 2000) and were purchased from Sigma-Aldrich (Taufkirchen, Germany). Control aphids were injected with equivalent concentrations of dsRNA encoding the insect metalloproteinase inhibitor IMPI (GenBank gbAY330624.1) from the greater wax moth *Galleria mellonella* (Clermont et al. 2004; Wedde et al. 2007). This sequence is not present in insects other than the Lepidoptera (Mylonakis et al. 2016).

We injected 8-day-old apterous L4 nymphs with ∼50 ng dsRNA in a total volume of 6.9 nl under a stereomicroscope using a Nanoliter 2000 injector with a Sys-Micro4 controller (World Precision Instruments, Berlin, Germany). Glass microcapillaries for injection were prepared using a PN-30 puller (Narishige International Limited, London, UK). Prior to injection, aphids were immobilized with their dorsal thorax on a vacuum holder (van Helden and Tjallingii 2000). The dsRNA was applied to aphids with an injection rate of 2 nl/s according to Mutti et al. (2006). The experiment was carried out three times, each replicate with 15 aphids per group.

## Quantification of HSP83 expression by real-time PCR

Total RNA was extracted from the aphids 1, 3, and 6 days after RNAi treatment. The 3 × 5 aphids per treatment were collected and RNA was extracted using Direct-zol™ RNA MiniPrep with TRI-Reagent® (Zymo Research, Freiburg, Germany). Complementary DNA was synthesized using 1 μg of total RNA, oligo(dT)18 primers, and the First Strand cDNA Synthesis Kit (Thermo Fisher Scientific, Waltham, MA) according to the manufacturer’s recommendations. Real-time PCR was performed on a StepOnePlus system (Applied Biosystems) using gene-specific TaqMan Gene Expression assays (Thermo Fisher Scientific). The assay was carried out according to the manufacturer’s protocol using custom TaqMan gene expression assays, including the HSP83 gene (GenBank XM_001943137.3) and the reference ribosomal protein L32 (rpl32) gene (GenBank NM_001126210.2). To ensure reproducibility, gene expression was tested in triplicate. Data were analyzed using the ΔΔCq method in REST (Pfaffl 2001; Pfaffl et al. 2002).

### Assaying longevity, fecundity, and embryogenesis

Survival assays and reproduction assays were conducted separately using 15 aphids per group in each test. Aphids placed on a leaf in an agar plate were checked each day and nymphs were removed. Plates were kept in a climate cabinet under the conditions described by Will and Vilcinskas (2015). Images of whole animals were taken 5, 7, and 12 days after injection (dai) using a MZ16FA stereo microscope (Leica, Wetzlar, Germany). Ovaries of four to five living aphids from each treatment (untreated, *impi* dsRNA and *hsp83* dsRNA) were dissected 12 days after injection in insect Ringer’s solution (9 g NaCl, 0.25 g MgCl_2_ × 6H_2_O, 0.2 g KCl, 1 g glucose in 1 l H_2_O, pH 6.8). Dissected ovaries were observed under a MZ16FA stereo microscope, and digital images were analyzed to determine the number of ovary follicles and their developmental stage according to Schmidtberg and Vilcinskas (2016).

### Image analysis for coloring and body plan area

The quality of images of whole animals and dissected ovaries was improved for brightness and contrast using Photoshop CS v5.1 (Adobe Systems Inc., San Jose, CA, USA). Images forming part of an image set (images from one experiment) were treated in the same manner. RGB images from adults/embryos were transformed to an 8-bit gray scale, pixel gray values were measured and a whole body/embryo mean was calculated using ImageJ v1.42q (Wayne Rosband, National Institute of Health, USA). To compensate for color changes in adults that naturally occur during the aging of aphids, relative brightness was calculated whereas the mean gray value of untreated control animals at each time point was set to 1. The gray value of embryos from untreated mothers was set to 1 as well. The relative brightness of adults/embryos of the microinjected control group (*impi* dsRNA) and HSP83-depleted aphids was calculated in relation to the gray value of untreated adults/embryos. We analyzed images of 15 untreated adult aphids and 14 adult dsRNA-injected aphids (*impi* dsRNA and *hsp83* dsRNA). Color determination of embryos was based on the measurement of nine late-stage embryos (embryo stage ≥18) from three different ovaries per treatment.

### Statistical analysis

Survival analysis was carried out using the Kaplan-Meier log-rank test in Sigma Plot v11. Reproduction, embryogenesis, coloring, and body plan area data were compared by analysis of variance (ANOVA). The level for statistical significance was set to *p* = 0.05.

## Results

### Effect of attenuated HSP83 expression on longevity and fecundity

**Fig. 1:**
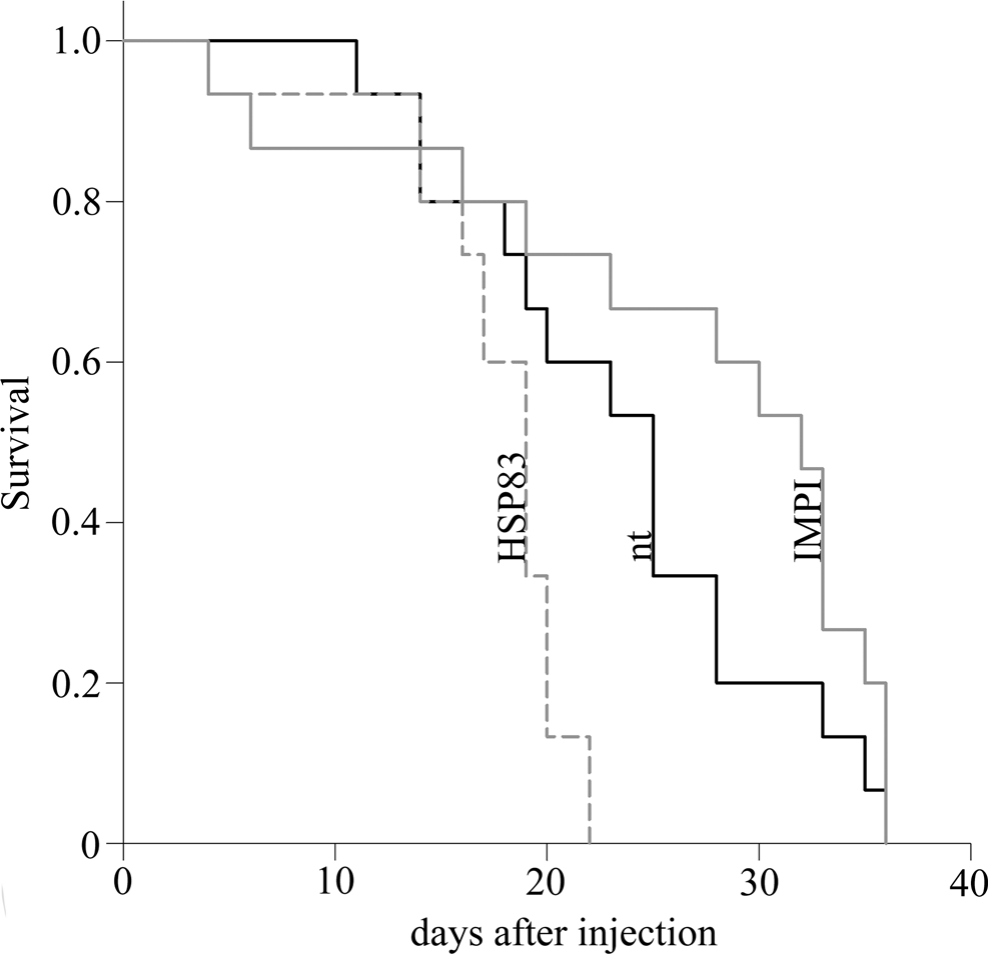
Survival analysis of aphids treated with *hsp83* dsRNA. HSP83-attenuated aphids were compared to control groups using the Kaplan-Meier log-rank test. Survival was significantly reduced for aphids in the *hsp83* dsRNA treatment group compared with untreated (nt) aphids (*p* < 0.01) and aphids injected with *impi* dsRNA (*p* < 0.001). There was no significant difference between the control groups (*p* > 0.05)

**Fig. 2:**
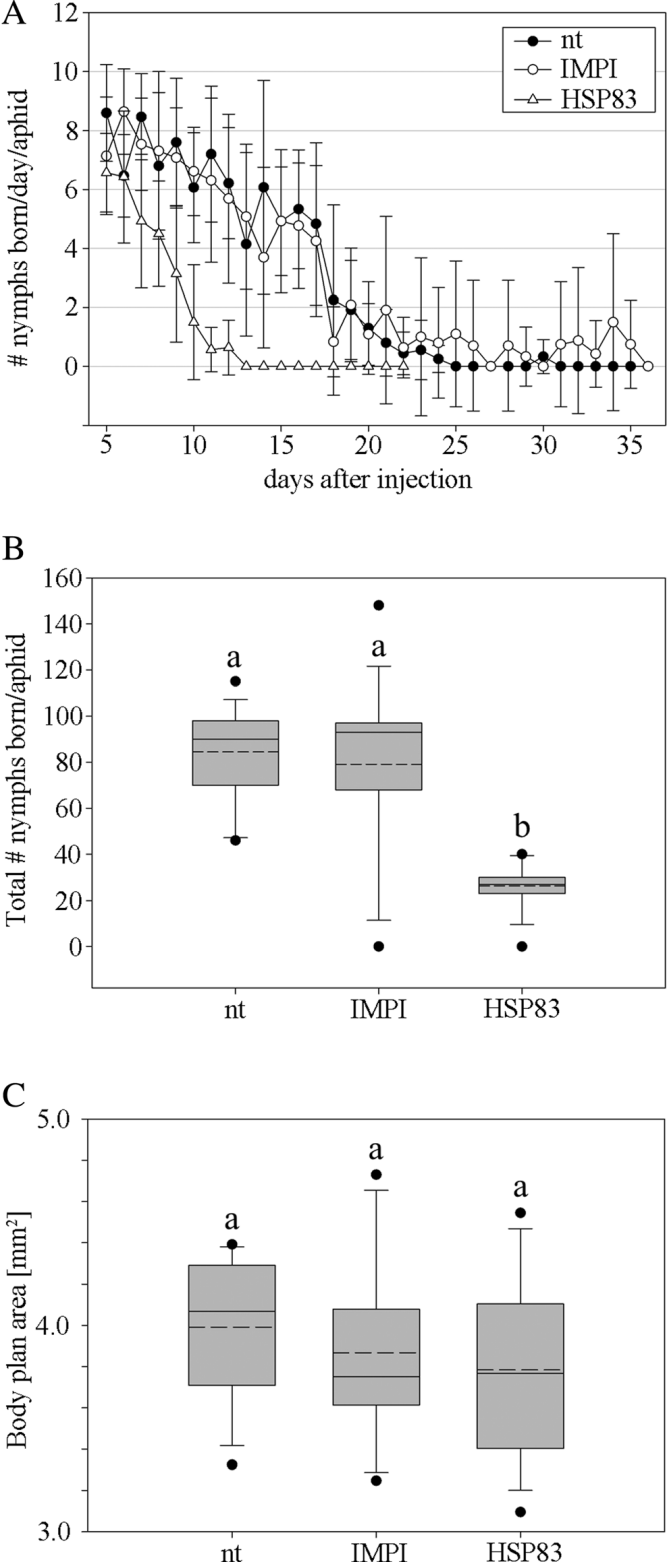
Influence of HSP83 attenuation on the reproduction of *A. pisum*. **a** Reproduction of aphids treated with *hsp83* dsRNA decreases more rapidly and ends at an earlier point compared to the control groups. **b** Lifetime reproduction of aphids treated with *hsp83* dsRNA is significantly reduced compared to both controls (*p* < 0.001). There was no significant difference between the control groups (*p* > 0.05). **c** The body plan area of aphids treated with *hsp83* dsRNA is marginally reduced but is not significantly affected compared with untreated (nt) aphids and aphids treated with *impi* dsRNA (*p* > 0.05). A significant difference between the groups is indicated in the graph by *different letters*

Kaplan-Meier log-rank of survival data from untreated viviparous *A. pisum* individuals was compared with those injected with dsRNA encoding either *hsp83* or an unrelated control gene, the insect metalloproteinase inhibitor *impi*, which is specific for lepidopterans (Mylonakis et al. 2016). Aphids in the untreated and the injected control groups survived for a maximum of ∼35 days, whereas those injected with *hsp83* dsRNA survived for a maximum of ∼22 days (Fig. 1). Depleted HSP83 expression in viviparous *A. pisum* individuals also significantly reduced the number of nymphs born per aphid and per day compared with the untreated and *impi* controls (Fig. 2). Aphids in the *hsp83* dsRNA group produced a mean of 27 nymphs during the experiment, which was significantly lower (*p* < 0.001) than both control groups. The same result emerged independently when we assessed the total number of nymphs born per aphid per day (Fig. 2a) or born per aphid throughput the experiment (Fig. 2b).

### Effect of attenuated HSP83 expression on aphid embryogenesis and eclosion

**Fig. 3:**
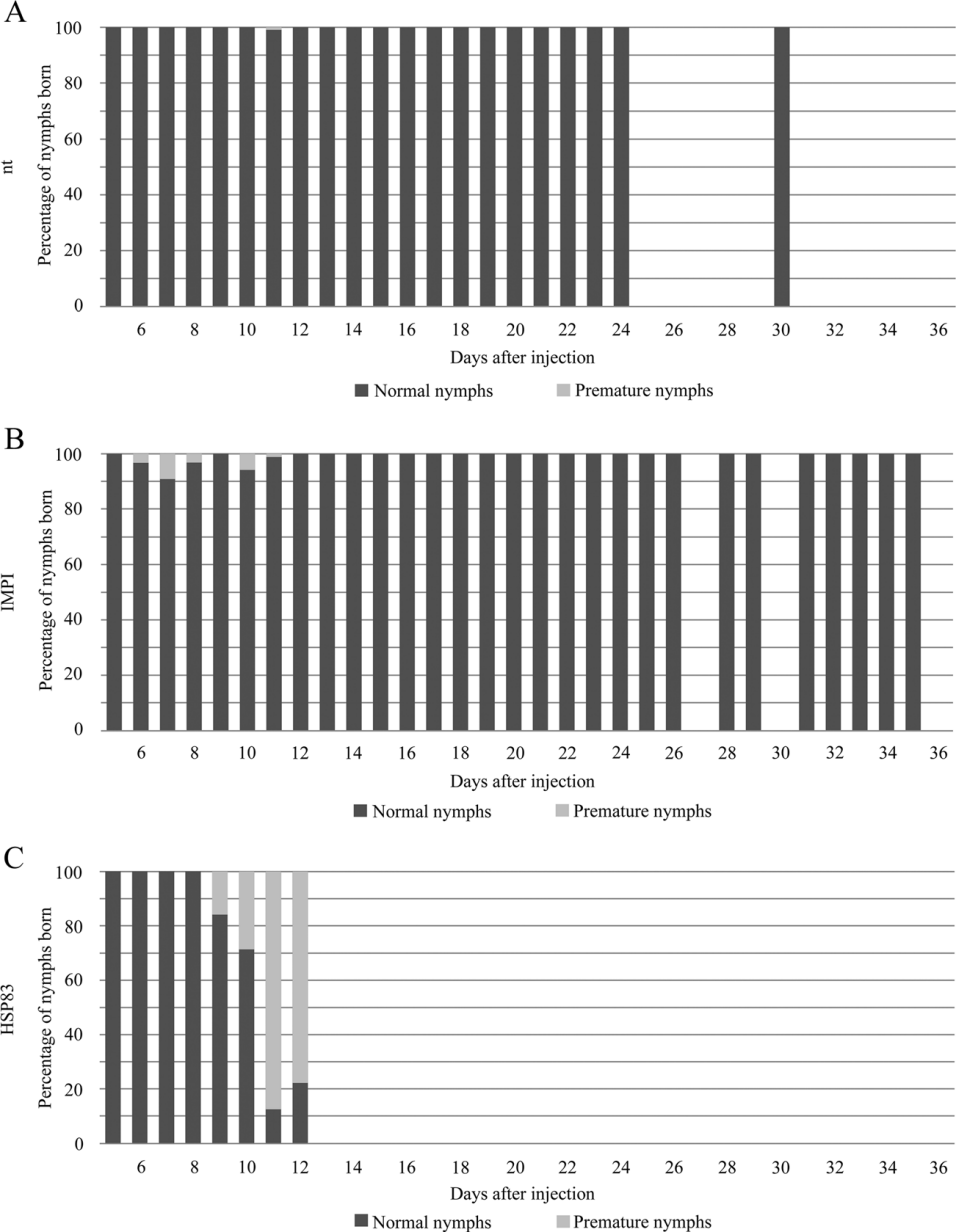
Impact of HSP83 attenuation on the percentage of premature nymphs. In comparison to the untreated control (**a**), control aphids injected with *impi* dsRNA (**b**) produce a small proportion (maximum 9 %) of premature nymphs 6–10 days after injection. In contrast, aphids injected with *hsp83* dsRNA (**c**) show a rapid increase in the percentage of premature nymphs from day 9 after injection until reproduction stopped (12 days after injection)

**Fig. 4:**
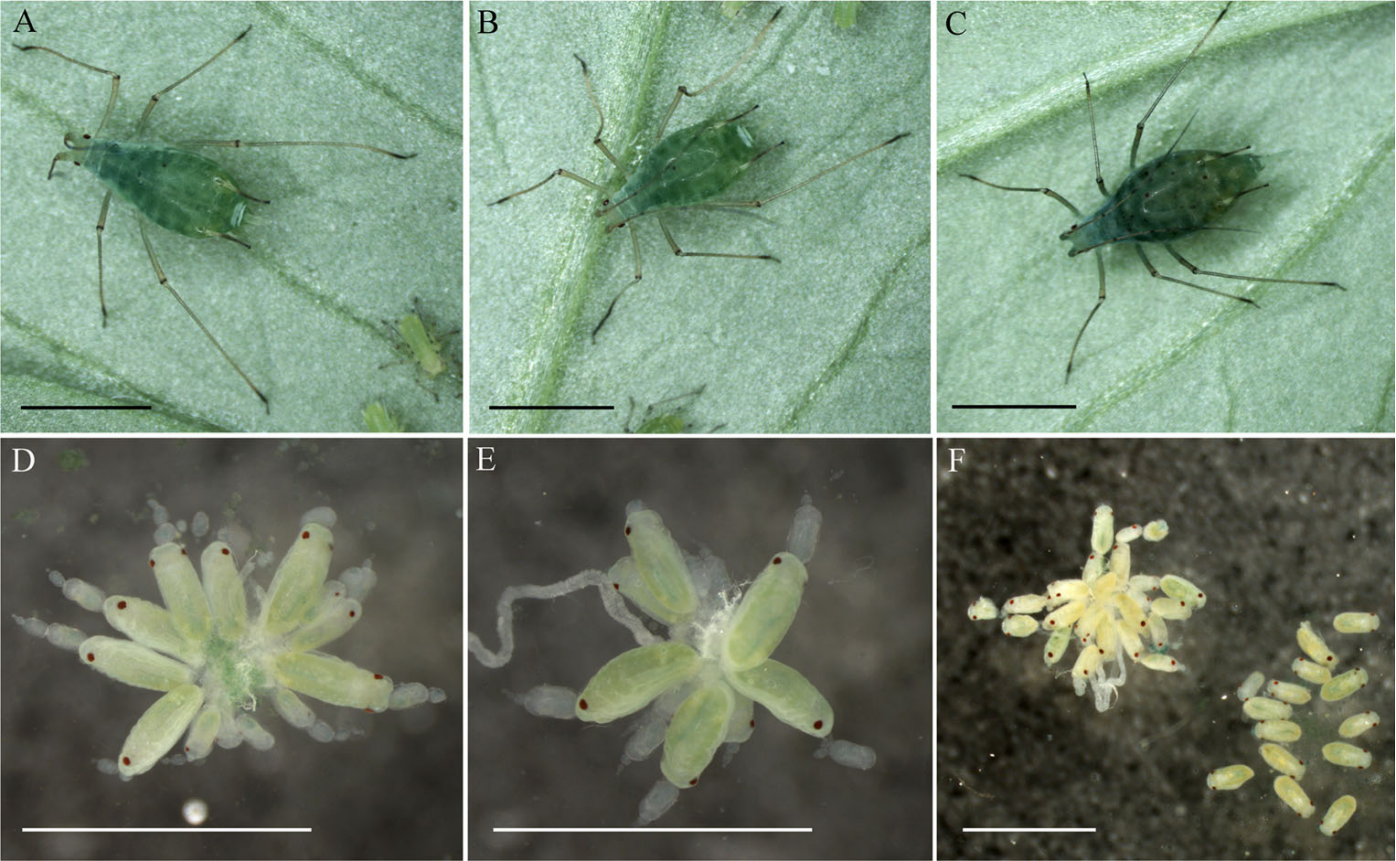
Influence of HSP83 attenuation on aphid phenotype. Untreated (**a**) and *impi* control (**b**) adult apterous female aphids are bright green, and a small number of embryo eye spots are visible through the cuticle. *Scale bars* = 2 mm. Aphids treated with *hsp83* dsRNA (**c**) are dark green, and more eye spots (*arrowheads*) can be seen compared to the control groups. *Scale bar* = 2 mm. Dissected ovaries of untreated (**d**) and *impi* control aphids (**e**) contain embryos at developmental stage 6 and earlier (see Table 1). *Scale bar* = 1 mm. In contrast, these developmental stages are absent in ovaries from aphids treated with *hsp83* dsRNA, and some late-stage embryos are not attached to ovaries (**f**). *Scale bar* = 1 mm

Using the gene-specific TaqMan Gene Expression assay, we observed attenuated HSP83 expression levels after 1 day, *hsp83* dsRNA. However, the attenuation of gene expression was not significant, and the expression increased slightly 3 and 6 days after the injection of (Supplementary Fig. S1). HSP83 depletion did not appear to affect L4 (8-day-old) nymphs, which developed into reproductive adults. However, the attenuated expression of HSP83 resulted in the eclosion of many premature nymphs (Fig. 3). These died a few hours after eclosion and their antennae and legs remained folded. In the untreated control group, 1 % of eclosed nymphs were premature and the phenomenon was only observed on day 11. In the control group injected with *impi* dsRNA, 2–9 % of eclosed nymphs were premature and the eclosions occurred between days 6 and 10 after injection. But in the group injected with *hsp83* dsRNA, the proportion of premature eclosed nymphs increased from 16 to ∼80 % between days 9 and 12 after treatment, the time during which the reproduction phase of the *hsp83* dsRNA-injected aphids is completed (Fig. 2a). Remarkably, when observing embryos through the integument of the ovary 12 dai, we observed many more translucent eyes representing developing embryos in the group treated with *hsp83* dsRNA compared to the two control groups (Fig. 4a–c). Dissection of ovaries from four *hsp83* dsRNA-injected adults and from five adult aphids from each control group revealed that aphids injected with *hsp83* dsRNA contained no embryos at or before developmental stage 6, whereas embryos were present in both control groups. In addition, the second developmental phase (embryo stages 7–13) differed significantly between the HSP83-depleted aphids and those from control groups. A striking characteristic of aphids treated with *hsp83* dsRNA was the presence of embryos at later developmental stages (embryo stage ≥18) that are detached from the ovarioles and lie free inside the hemocoel (Fig. 4f). These are not present in either of the control groups (Table 1; Fig. 4d, e). Effects were only considered to be HSP83-dependent when significant differences were observed between the HSP83-depleted aphids and both control groups.

**Table 1:** Comparative analysis of parthenogenetic embryo development in HSP83-attenuated aphids and control groups

	nt	IMPI	HSP83	*p* value nt vs. IMPI	*p* value nt vs. HSP83	*p* value IMPI vs. HSP83
Stage ≤6	5.00 ± 4.12	2.40 ± 1.14	0	0.211	0.048	0.004
Stages 7–13	7.60 ± 3.65	13.00 ± 5.29	2.50 ± 2.38	0.097	0.047	0.008
Stages 14–17	1.80 ± 2.49	5.80 ± 2.68	0.75 ± 0.96	0.040	0.456	0.009
Stage ≥18	9.40 ± 3.21	5.80 ± 1.30	18.25 ± 10.81	0.049	0.121	0.036
Free stage ≥18	0	0	7.25 ± 4.99	–	0.002	0.002
Total	23.80 ± 8.64	27.00 ± 6.32	21.50 ± 11.09	0.523	0.736	0.377

The number of embryos is given together with the standard deviation (mean ± SD) for developmental phases for each of the treatments. Statistical analysis was performed by ANOVA and the corresponding *p* values are presented

### Effect of attenuated HSP83 expression on aphid color

**Fig. 5:**
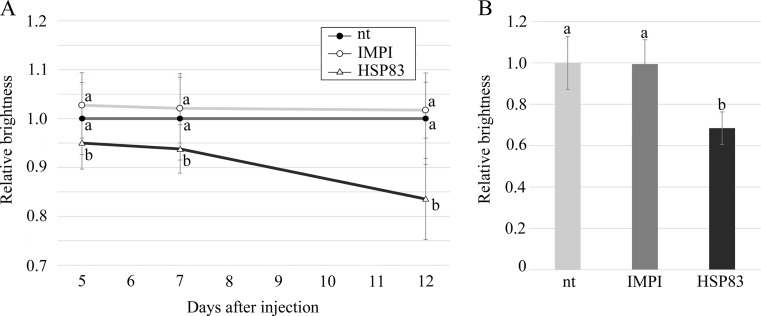
Change of coloring of HSP83-attenuated aphids and their embryos. Adult apterous aphids (**a**) show a darkening color in response to HSP83 silencing over time. **b** Embryos of HSP83-attenuated aphids (12 dai) show also a significantly darker coloring than embryos of control groups. A significant difference (*p* ≤ 0.05) between the groups is indicated in the graph by *different letters*

The attenuated expression of HSP83 caused the injected adults (Fig. 5a) to become significantly darker in color (Fig. 4c) during the observation period, with a mean relative brightness of 0.95 (5 dai; *p* = 0.047), 0.94 (7 dai; *p* = 0.024), and 0.84 (12 dai; *p* = 0.002), compared to untreated aphids whose relative brightness was set to 1 at each time point. This was also observed for the embryos (Fig. 5b) inside HSP83-attentuated adults, with a mean relative brightness of 0.68 (12 dai; *p* < 0.001). Between the control groups of untreated and *impi* dsRNA-injected animals, there were no significant differences in coloring at any time point for adults (5 dai: *p* = 0.31; 7 dai: *p* = 0.488; 12 dai: *p* = 0.582) or embryos (12 dai: *p* = 0.161).

## Discussion

The postulated evolutionary and ecological role of HSPs predicts that their expression is induced by exposure to environmental stressors and influences fitness parameters such as lifespan and fecundity (Sorensen et al. 2003). However, exposure to mild heat shock or microbial elicitors of immune responses only moderately induced the expression of *hsp90* and its homolog *hsp83* in the model insects *T. castaneum* (Freitak et al. 2012) and *A. pisum* (Gerardo et al. 2010). The HSP90 family also plays a role in insect spermatogenesis, oogenesis, and embryogenesis (Ding et al. 1993; Yue et al. 1999; Song et al. 2007). The developmental roles of HSP90 have recently expanded beyond those known in embryogenesis to encompass functions in post-embryonic development such as the regulation of compound eye formation (Knorr and Vilcinskas 2011). As in the latter study, we also used RNAi-mediated attenuation of HSP expression to explore the functions of *hsp83* in hemimetabolous aphids, which have evolved a peculiar life cycle combining the alternation of sexual and asexual reproduction with an unusual (autosome-like) inheritance of the X chromosome (The International Aphid Genomic Consortium 2010). In accordance with our expectations, we found that the injection of *hsp83* dsRNA into *A. pisum* reduced the lifespan, fecundity, and number of viviparous offspring, even though the attenuation of HSP83 expression was not significant. The confirmation of gene knockdown in RNAi experiments is sometimes difficult, particularly if the target gene is expressed at a low level, because it depends on the selected reference genes (Holmes et al. 2010; Baumann et al. 2015).

The negative impact of attenuated HSP83 expression on the survival of *A. pisum* (Fig. 1a) appears to be in striking agreement with the role of the homologous HSP90 in the longevity of *D. melanogaster* (Chen and Wagner 2012) suggesting that at least one function of HSP83 is evolutionarily conserved in insects. The proposed role of HSP90 in the fecundity of *D. melanogaster* (Chen and Wagner 2012) was also observed in *A. pisum*, where attenuated HSP83 expression significantly inhibited the formation of viviparous offspring compared to untreated controls and controls treated with *impi* dsRNA. Reduced HSP83 expression also increased dramatically the number of immature eclosed nymphs (Fig. 3). This suggests that HSP83 displays a previously unknown role in embryogenesis. The low number of early-stage embryos in the ovaries of aphids injected with *hsp83* dsRNA is presumably caused by the resorption of embryos, occurring under suboptimal environmental conditions, allowing the late-stage embryos to reach maturity (Ward and Dixon 1982).

Members of the HSP90 family are known to participate in signal transduction (Nollen and Morimoto 2002), e.g., by activating steroid receptors (Bohen and Yamamoto 1993). We therefore propose that HSP83 expression regulates embryogenesis and eclosion, which are both strongly influenced by environmental factors (Ward and Dixon 1982; Altincicek et al. 2008). Its function may be mediated by the recently reported interaction with the transcription factor Broad Z7 because Cai et al. (2014) reported that HSP90 associates with the Broad Complex/Tramtrack/Bric-a-brac domain of Broad Z7 to prevent its degradation in the moth *Helicoverpa armigera*, and Piulachs et al. (2010) showed that Broad plays key roles in embryogenesis of the cockroach *Blattella germanica*. Therefore, it appears plausible that the downregulation of *hsp83* expression to below a specific but unknown threshold could disrupt the interplay between embryonic development and eclosion, leading to the presence of embryos that are detached from the ovarioles and lie free inside the hemocoel of *hsp83* dsRNA-injected aphids (Fig. 4f). Interestingly, the observed impact of injected *hsp83* dsRNA on embryos suggests the occurrence of parental RNAi because it has been reported that injected or orally delivered dsRNA can cause trans-generational attenuation of gene expression in aphids (Abdellatef et al. 2015).

The diverse roles of HSP83 in aphid longevity, fecundity, and embryogenesis may reflect either a distinct pool of client proteins that interact with HSP83 (Erlejman et al. 2014) or the requirement for specific co-chaperones to achieve appropriate HSP83 targeting (Johnson 2012). Multiple isoforms and transcript variants of HSP90 family members such as HSP83, which have been identified in *A. pisum* and *Myzus persicae* (cf. AphidBase), appear to act as chaperones for different types of client proteins related to longevity, fecundity, and development (Haslbeck et al. 2012).

Interestingly, the darker color of adult aphids and their embryos in the *hsp83* dsRNA group concurs precisely with the proposed epigenetic role of this chaperone in the protection of insects against environmental stress imposed by UV-A (Sang et al. 2012) or heat (Gilbert et al. 2007). Temperature acts on melanin production by modulating a chromatin regulator network, interacting genetically with the transcription factor Bric-a-brac, which is also an HSP83 target (Cai et al. 2014). HSP90 in *D. melanogaster* and in mammals can target paused RNA polymerases to activate genes in response to environmental stimuli (Sawarkar et al. 2012). Our data suggest that the aphid HSP83 homolog may have a related function.

The collection of altered phenotypes observed in *A. pisum* following the RNAi-mediated attenuated expression of *hsp83* can be explained by an alternative hypothesis based on the occurrence buffered phenotypic variation in response to environmental stimuli. The silencing of HSP90 in *D. melanogaster* resulted in transposon-mediated mutagenesis (Specchia et al. 2010). However, further research is required to confirm whether the attenuation of HSP83 expression in *A. pisum* also induces the mobilization of transposable elements, ultimately causing the observed phenotypic variation.

In conclusion, attenuated HSP83 expression in the hemimetabolous aphid *A. pisum* has revealed functional similarities and differences compared with its reported roles in holometabolous insects such as *T. castaneum* (Knorr and Vilcinskas 2011). The observed negative impact of reduced HSP83 expression on aphid survival and its complex effects on reproduction and embryogenesis suggest that the protein has pleiotropic roles involving the mediation of environmental stimuli affecting these complex parameters. The resulting functional plasticity could be achieved by targeting different client proteins, by recruiting distinct co-chaperones, or by inducing transposon-mediated mutagenesis. The entity of our results implicates that HSP83 represents another promising target for RNAi-mediated approaches aiming the engineering of aphid-proof crops (Will and Vilcinskas 2013; Abdellatef et al. 2015).

## Electronic supplementary material

Fig. S1 (DOCX 273 kb)

## Abbreviations

dsRNA: double-stranded RNA
HSP: heat shock protein
RNAi: RNA interference dai
