# Supplementary material for: Heat shock protein 83 plays pleiotropic roles in embryogenesis, longevity, and fecundity of the pea aphid Acyrthosiphon pisum
Source: Dev Genes Evol. 2016 Oct 14;227(1):1–9. doi: 10.1007/s00427-016-0564-1 (PMC5203865; doi:10.1007/s00427-016-0564-1)
Supplement: Supplementary file 1 — (DOCX 273 kb) [file 427_2016_564_MOESM1_ESM.docx]

**Heat shock protein 83 plays pleiotropic roles in embryogenesis, longevity and fecundity of the pea aphid *Acyrthosiphon pisum***

**Torsten Will**^1,2^, **Henrike Schmidtberg**^1^, **Marisa Skaljac**^3^**, Andreas Vilcinskas**^1,3^*****

**^1^**Institute of Insect Biotechnology, Justus-Liebig-University of Giessen, Heinrich-Buff-Ring 26-32, D-35392 Giessen, Germany

^2^Current address: Institute of Phytopathology, Justus-Liebig-University of Giessen, Heinrich-Buff-Ring 26-32, D-35392 Giessen, Germany

**^3^**Fraunhofer-Institute for Molecular Biology and Applied Ecology (IME) Project Group ‘Bioresources’ Winchesterstraße 2, D-35394 Giessen, Germany

**^*^**Author for correspondence: Andreas Vilcinskas [andreas.vilcinskas@agrar.uni-giessen.de](mailto:andreas.vilcinskas@agrar.uni-giessen.de), Phone: +49 (0)641 99 37600; Fax: +49 (0)641 99 37609

**
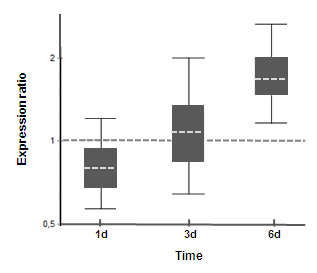
**

Supplementary Figure 1. HSP83 gene expression in the RNAi experiment. Expression of the HSP83 gene after normalization with the rpl32 reference gene in pea aphid samples after one, three and six days after RNAi. The dashed grey line indicates the boundaries of HSP83 mRNA level variation in the control group. d, day.
